# Heterogeneity of Breast Cancer Associations with Common Genetic Variants in *FGFR2* according to the Intrinsic Subtypes in Southern Han Chinese Women

**DOI:** 10.1155/2015/626948

**Published:** 2015-09-03

**Authors:** Huiying Liang, Xuexi Yang, Lujia Chen, Hong Li, Anna Zhu, Minying Sun, Haitao Wang, Ming Li

**Affiliations:** ^1^School of Biotechnology, Southern Medical University, Shatai Southern Road 1023, Baiyun District, Guangzhou, Guangdong 510515, China; ^2^Institute of Pediatrics, Guangzhou Women and Children Medical Center, Jinsui Road 9, Tianhe District, Guangzhou, Guangdong 510623, China; ^3^Breast Center Nanfang Hospital, Southern Medical University, Shatai Southern Road 1023, Baiyun District, Guangzhou, Guangdong 510515, China; ^4^Department of Primary Public Health, Guangzhou Center for Disease Control and Prevention, Qide Road 1, Baiyun District, Guangzhou, Guangdong 510440, China; ^5^Obstetrics Outpatient Clinic, Guangzhou Women and Children Medical Center, Jinsui Road 9, Tianhe District, Guangzhou, Guangdong 510623, China

## Abstract

GWAS have identified variation in the *FGFR2* locus as risk factors for breast cancer. Validation studies, however, have shown inconsistent results by ethnics and pathological characteristics. To further explore this inconsistency and investigate the associations of *FGFR2* variants with breast cancer according to intrinsic subtype (Luminal-A, Luminal-B, ER−&PR−&HER2+, and triple negative) among Southern Han Chinese women, we genotyped rs1078806, rs1219648, rs2420946, rs2981579, and rs2981582 polymorphisms in 609 patients and 882 controls. Significant associations with breast cancer risk were observed for rs2420946, rs2981579, and rs2981582 with OR (95% CI) per risk allele of 1.19 (1.03–1.39), 1.24 (1.07–1.43), and 1.17 (1.01–1.36), respectively. In subtype specific analysis, above three SNPs were significantly associated with increased Luminal-A risk in a dose-dependent manner (*P*_trend_ < 0.01); however, only rs2981579 was associated with Luminal-B, and none were linked to ER−&PR− subtypes (ER−&PR−&HER2+ and triple negative). Haplotype analyses also identified common haplotypes significantly associated with luminal-like subtypes (Luminal-A and Luminal-B), but not with ER−&PR− subtypes. Our results suggest that associations of *FGFR2* SNPs with breast cancer were heterogeneous according to intrinsic subtype. Future studies stratifying patients by their intrinsic subtypes will provide new insights into the complex genetic mechanisms underlying breast cancer.

## 1. Introduction

Recently, large genome-wide association studies (GWAS) have identified nearly 70 genetic susceptibility loci associated with breast cancer risk [1–7]. Specifically, a locus within an intron of the* FGFR2* gene is consistently the most strongly associated one [1, 3, 8]. As the majority of GWAS were performed mainly in Caucasian populations, replicate studies have often failed to extrapolate the association to diverse ethnic regions, such as Asians [9] and African-Americans [10]. These inconsistencies mainly stem from differences in linkage disequilibrium (LD) patterns and variable minor allele frequencies of SNPs between ethnicities according to the abovementioned studies.

From another point of view, breast cancers vary greatly in clinical behavior, morphological appearance, and molecular alterations. Genomic studies have established that breast cancer can be divided into 4 major intrinsic subtypes (Luminal-A, Luminal-B, HER2-enriched, triple negative) that differ significantly in terms of incidence, survival, and response to therapy [11, 12]. Therefore, determining whether genetic risk factor associations for breast cancer differ by subtype of the tumors represents a critical etiologic question. Evidence that genetic variants in* FGFR2* may influence tumor subtype is provided by the fact that susceptibility loci in* FGFR2* have stronger associations for estrogen receptor positive disease (ER+) than ER− [13].

However, the first wave of GWAS has been conducted with a predominance of ER positive disease and is unable to determine whether tumor subtypes modify the association between breast cancer risk and the susceptibility loci recently identified. Additionally, recent interest has focused on ER expression status [4, 14–16] and few of these studies have provided data for subtypes defined jointly by ER, progesterone receptor (PR), and human epidermal growth factor receptor 2 (HER2) status or more biomarkers [17]. Determining whether breast cancer genetic risk factors are linked to tumors with specific intrinsic subtypes may provide a gateway for developing tailored prevention and early detection strategies.

We therefore decided to use the data source provided by the Southern China Breast Cancer Genetics Study (SCBCGS) to evaluate the hypothesis that tumor intrinsic subtypes, in particular those defined jointly by the expression of ER, PR, HER2, and Ki-67, modify the association between breast cancer risk and the common* FGFR2* intron-2 polymorphisms recently identified. Without a doubt, this paper will expand and refine our previous reports on analyses only by ER status through including up to three additional tumor markers. Moreover, the detailed analyses are allowing us to verify whether definition of the genomic intrinsic subtypes of breast cancer can provide another window into the underlying heterogeneity between different studies and thus make more definite conclusions than previous reports.

## 2. Materials and Methods

### 2.1. Study Population

Individuals included in the current analysis were Han Chinese women who participated in the SCBCGS. The SCBCGS was a multicenter, hospital-based study of breast cancer conducted among Han Chinese women from three areas of the Southern China, including Canton, Chongqing, and Nanchang. Previous reports have described the study population and enrollment process in detail [18–21]. Briefly, consecutive patients with histologically confirmed primary breast cancer were recruited from defined hospitals. Control individuals were selected using the Resident Registry of each city and were frequency matched on ethnicity, age (±5 years), and community of residence to the cases. Detailed information on histories of menstrual and reproductive factors, hormone therapy (HT), weight, height, and family history of breast cancer for each participant was collected during in-person interviews.

Extensive early studies confirmed that late age of first term pregnancy (>30 years) and early menarche (<13 years) are known risk factors for breast cancer [22, 23]. Thus, we included age at menarche and first-term pregnancy in our multivariable models. In addition, to determine the heterogeneity of relationship between variants in FGFR2 and sporadic breast cancer by intrinsic subtypes, only patients without family history of breast cancer were eligible for present study.

The study was approved by the institutional review boards at all participating institutes (IRB numbers 2009-SCBCGS-GZ-01, 2009-SCBCGS-CQ-01, and 2009-SCBCGS-NC-01), and all participants provided written, informed consent before participating in the study.

### 2.2. DNA Extraction and Genotyping

Laboratory protocols for the DNA extraction and genotyping methods used by the SCBCGS have been previously described in detail [18, 19]. Briefly, genomic DNA was extracted from whole blood using TIANamp Genomic DNA Purification Kit according to the manufacturer's protocol and stored at −80°C until used for further analysis.

Recent GWAS have confirmed that all the significantly associated SNPs of FGFR2 with breast cancer risk fell in a 25 kb linkage disequilibrium (LD) block entirely within intron-2. To further replicate the GWAS findings in the Chinese women, we first identified 7 variants showing association with breast cancer in one or more GWAS [1, 3, 8]. Given the sample size and statistical power of present study, two SNPs (rs7895676 and rs11200014) were excluded because of a low minor allele frequency (MAF) of less than 25% in Han Chinese from Beijing (CHB) from HapMap. Thus, only 5 SNPs (rs1078806, rs1219648, rs2420946, rs2981579, and rs2981582) were selected for analysis in this study. The genotyping of SNPs was done using the SEQUENOM MassARRAY matrix-assisted laser desorption ionization time of flight mass spectrometry platform [18, 19].

### 2.3. Classification of Biologic Subtype

**Figure 1:**
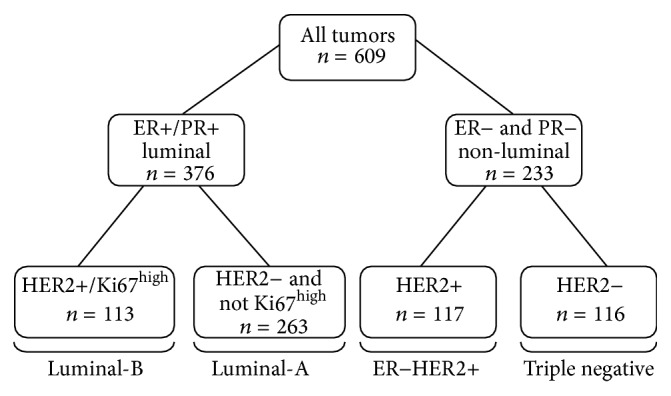
Classification of breast cancer tumors according to the expression of ER, PR, HER2, and Ki67 (tumor subtype nomenclature explanation: / = “and/or,” & = “and”).

Finally, database review identified 609 eligible patients with details on ER, PR, HER2, and Ki67 expression. Four subtypes were constructed: (i) triple negative (ER−, PR−, and HER2−), (ii) ER−HER2+ (ER−, PR−, and HER2+), (iii) Luminal-B (ER+ and/or PR+ and either HER2+ and/or Ki67^high^), and (iv) Luminal-A (ER+ and/or PR+ and not HER2+ or Ki67^high^).  Figure 1 shows the classification scheme based on combinations of the biomarker. ER and PR were considered positive if immune-histochemistry (IHC) staining was ≥10%; an IHC score of 3+ or HER2 amplification by fluorescence-in-situ-hybridization (FISH) score was used to determine HER2 positive status [24]. At a Ki67 cutoff point of ≥10%, tumors were designated “high proliferation” [25].

### 2.4. Statistical Analysis

For each SNP, deviation of genotype frequencies in controls from the Hardy-Weinberg-Equilibrium (HWE) was assessed by a goodness-of-fit *χ* ^2^ test. Differences in frequencies of SNP alleles and genotypes between cases and controls were evaluated using chi-square test or Fisher's exact test as appropriate. Breast cancer risk was estimated as odds ratios (ORs) and 95% confidence intervals (CIs), based on unconditional logistic regression and adjusted for potential confounders. Analyses were carried out assuming a dominant, codominant, and additive allelic effect for each polymorphism. The Cochran-Armitage trend test was performed to test additive genetic effect model.

Linkage disequilibrium (LD) pattern and population haplotype frequencies for the SNPs were estimated using the online SNPStats tool using an expectation maximization algorithm [26]. Using the most frequent haplotype as the reference group, an additive model was used to introduce haplotype counts, and an unconditional regression model was applied to calculate ORs (95% CIs) adjusting for potential confounders.

Stratified analysis according to intrinsic subtypes was additionally conducted. All statistical tests were two-sided, and *P* < 0.05 was considered significant. To correct multiple testing, we estimated the adjusted significance by applying the Bonferroni correction for all the SNPs tested in the analysis. Statistical analysis was performed using SPSS version 19.0 (IBM SPSS Statistics for Windows, IBM Corporation, Somers, NY) unless otherwise specified.

## 3. Results

### 3.1. Characteristics of Controls and Cases

Table 1 shows the specific characteristics of the controls and cases by the intrinsic subtype. Compared with controls, cancer cases were older and more likely to be parous with first full-term pregnancy at ≥30 years and postmenupausal HT non-user. Notably, no significant differences were seen in basic characteristics between subtypes. Thus, age, HT use, menopausal status, and age at first full-term pregnancy were selected as potential confounders in the primary analyses.

### 3.2. Hardy–Weinberg Equilibrium Testing

The minor allele frequencies of all tested SNPs are roughly similar with the corresponding frequencies of the HCB (Chinese) and JPT (Japanese) population. All the observed genotype frequencies were found to be in agreement with HWE in controls except for rs1219648, which deviates from HWE (*P* < 1 × 10^−4^) and thus was excluded from the subsequent analyses (Table 2).

### 3.3. Associations with Breast Cancer Risk Overall and by Subtype Separately

Table 3 shows the allele and genotype distributions of the remaining four polymorphisms in the combined sample and in the subgroups. Chi-square test depicted significant associations for rs2420946 and rs2981579 with overall breast cancer risk (*P* = 3.4 × 10^−2^ and 1.4 × 10^−2^, resp.). After adjusting for the abovementioned potential confounders, logistic regression analysis further confirmed these associations which remained significant in dominant model for rs2420946 (C/T + T/T: *P* = 7.2 × 10^−3^), in both codominant (T/T: *P* = 5.0 × 10^−3^) and dominant (T/C + T/T: *P* = 8.1 × 10^−3^) model for rs2981579, and in per-allele model for rs2981579/T even after Bonferroni correction (*P* < 1.0 × 10^−2^).

In a subgroup of Luminal-A, the association between rs2420946 and breast cancer risk was the strongest (adjusted OR = 1.69, 95% CI: 1.13–2.53 for the T/T genotype and adjusted OR = 1.55, 95% CI: 1.12–2.15 for the C/T genotype compared with the common homozygote CC) in a dose-dependent manner (*P*_trend_ = 5.6 × 10^−3^). Significant associations were also observed between Luminal-A breast cancer risk and the homozygous minor allele genotype (T/T) for rs2981579 (adjusted OR = 1.68, 95% CI: 1.15–2.45, *P* = 7.0 × 10^−3^) and rs2981582 (OR = 2.01, 95% CI: 1.35–3.01, *P* = 1.0 × 10^−3^). However, after Bonferroni correction, only rs2420946 (*P*_correction_ = 1.3 × 10^−2^) and rs2981582 (*P*_correction_ = 3.4 × 10^−2^) were found to be associated with Luminal-A breast cancer risk under dominant model.

Under dominant model, rs2981579 was associated with increased Luminal-B breast cancer risk (*P* = 7.4 × 10^−3^; Table 3) with Bonferroni-adjusted *P* = 3.7 × 10^−2^. Rs2981582 showed a marginal association (*P* = 4.3 × 10^−2^) with ER−HER2+ breast cancer risk under dominant model. However, based on the multiple hypothesis testing, this association was not significant (Bonferroni-adjusted *P* = 2.2 × 10^−1^). No significant associations between selected SNPs and triple negative breast cancer risk were detected under any of the assumptions (Table 3).

### 3.4. Linkage Disequilibrium and Haplotype Analysis

**Figure 2:**
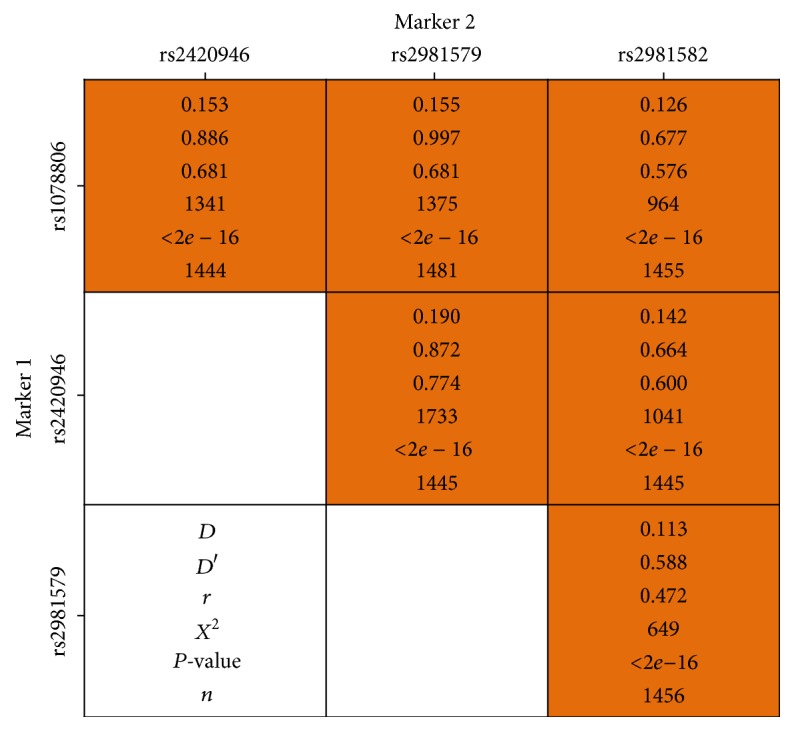
Linkage disequilibrium analyses of the four FGFR2 SNPs among Southern Han Chinese women.

**Figure 3:**
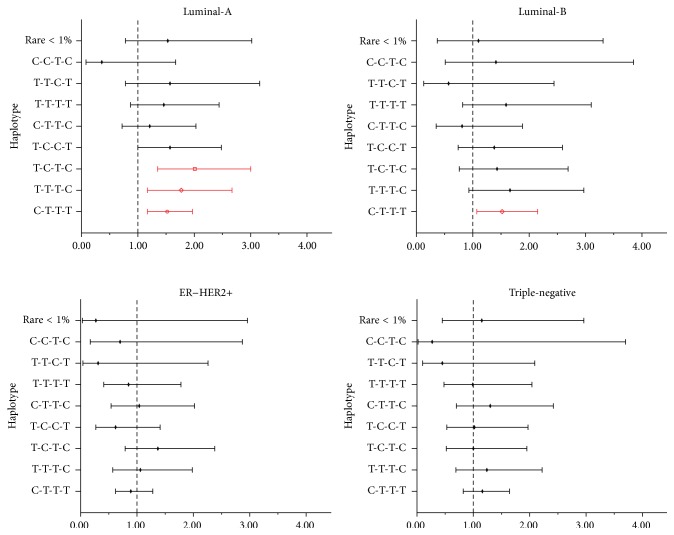
Associations of haplotypes of* FGFR2* rs1078806, rs2420946, rs2981579, and rs2981582 with breast cancer by subtype (*x*-axis: OR adjusted for age, age at first full-term pregnancy, menopausal status, and hormonal therapy status; horizontal bars showing 95% CIs.).

LD analyses showed that four variants were in moderate LD with each other (pairwise *r* ^2^ value range from 0.472 to 0.774 and *D*′ value range from 0.588 to 0.997) (Figure 2). Estimated haplotype (rs1078806-rs2420946-rs2981579-rs2981582) frequencies are presented in Table 4. Compared with the most common haplotype T-C-C-C, the C-T-T-T haplotype, with a frequency of 22.9% and containing three risk alleles (rs2420946T, rs2981579T, and rs2981582T), was associated with an increased breast cancer risk in the whole sample (adjusted OR = 1.30, 95% CI: 1.07–1.57, *P* = 8.1 × 10^−3^) (Table 4) and in subtypes Luminal-A (adjusted OR = 1.52, 95% CI: 1.17–1.97, *P* = 1.5 × 10^−3^) and Luminal-B (OR = 1.52, 95% CI: 1.07–2.15, *P* = 2.0 × 10^−2^) (Figure 3). Breast cancer risk, particularly Luminal-A breast cancer risk, was also significantly increased for carriers of haplotypes of T-T-T-C and T-C-T-C (Table 4 and Figure 3).

## 4. Discussion

*FGFR2* belongs to the FGFR family of tyrosine kinase receptors involved in various signaling pathways that contribute to the process of tumorigenesis through cell growth, apoptosis, and differentiation [27]. Subsequent analyses support their functional relevance to breast cancer risk that* FGFR2* polymorphisms located in intron-2 alter the binding of two transcription factors, Oct-1/Runx2 and C/EBPb, resulting in an increase of* FGFR2* gene expression both in cell lines and in breast tissue [28]. Specifically, a number of case-control studies have been conducted to investigate the association between* FGFR2* polymorphisms located in intron-2 with breast cancer susceptibility in Chinese populations [29–32]. However, these studies have yielded inconsistent results.

To investigate this inconsistency, one important step is to study whether these common variants interact with known breast cancer intrinsic subtypes. Thus, present study investigated whether 5 common* FGFR2* SNPs were associated with specific tumor subtypes defined by four markers. This will be the first Chinese study to validate and provide convincing evidence for heterogeneity in the strength of the association of* FGFR2* susceptibility locus with the risk of specific subtype. Furthermore, stratification of tumors also provided further insights into etiological heterogeneity.

First, this study confirmed that three SNPs in the second intron of* FGFR2* (rs2420946, rs2981579, and rs2981582) were significantly associated with increased risk of breast cancer, which validates earlier GWAS results [3]. This result is in accordance with Raskin et al. [33], who in Ashkenazi and Sephardi Jews population found statistically significant differences between breast cancer cases and healthy controls for rs2420946 and rs2981579 polymorphisms. Furthermore, Fu et al. [34] reported that rs2420946 polymorphism in the second intron of the* FGFR2* gene is significantly associated with increased breast cancer risk in nonfamilial breast cancer but not in familial breast cancer in a Chinese Han population. Similarly, in our study, to explore the genetic risk factors for sporadic breast cancer, all subjects also lacked a family history of cancer and free of other malignant diseases. Therefore, it is likely that some polymorphisms in intron-2 of* FGFR2* play a role in tumorigenesis in this subgroup of Chinese women.

Further subtype stratification analyses showed that rs2981579 was associated with the increased risk of both Luminal-A and Luminal-B according to dominant or codominant polygenetic risk models. Consistent with previous reports, this study confirmed that rs2420946 [35] and rs2981582 [36] are most strongly associated with Luminal-A, with no evidence for an association with the risk of triple negative tumors or ER−HER2+ tumors. As a receptor tyrosine kinase, above strong associations of* FGFR2* with luminal-like tumors are also consistent with the involvement of* FGFR2* in estrogen-related breast carcinogenesis [37].

In haplotype analysis, the risk haplotype of* FGFR2* (rs1078806C-rs2420946T-rs2981579T-rs2981582T) was associated with a significantly increased luminal-like breast cancer risk compared with the rs1078806T-rs2420946C-rs2981579C-rs2981582C haplotype, with no association observed for ER− and PR− tumors. Our findings on haplotype analysis are to several extents similar to previous studies due to further stratification by adding other tumor markers, not included in previous publications [29, 34, 38]. Furthermore, LD pattern between the four* FGFR2* variants in our Southern Chinese Han population was moderate, in contrast to Caucasians from the HapMap CEU samples (*r* ^2^ range from 0.97 to 1.0), but resembling other Asian populations [38], indicating a fairly independent risk effect of each locus in Asian population, but the results warrant screening in larger sample sets.

Therefore, different patterns of association with specific tumor subtypes observed in our study strengthen the evidence for hypothesis that genetic factors differ by intrinsic subtype. Therefore, different patterns of association with specific tumor subtypes observed in our study strengthen the evidence for hypothesis that genetic factors differ by intrinsic subtype [39]. To some extent, one study including cases unselected for intrinsic subtype status could result in contrary results and subsequent inconsistent conclusions. On the other hand, future studies stratifying patients by their intrinsic subtypes or including more homogenous tumor types will give much more power to classic case control studies.

One strength of present study was that ER, PR, HER-2, and ki67 status were all assessed using the same processing protocols and criteria for pathology review for all cases. However, several limitations of this study must be considered. First, though this current study has sufficient power (>90%) to detect a log-additive OR of 1.30 with allele frequencies >27%, providing sufficient sensitivity to detect most of the SNPs at the significance level of 0.05 (two-sided), other SNPs with ORs < 1.3 may be affected by insufficient power afforded in this study. Furthermore, the exact powers of three SNPs (rs2420946, rs2981579, and rs2981582) with Luminal-A and rs2981579 with Luminal-B were 79.64%, 72.46%, 92.15%, and 66.13%, respectively. However, we could not confirm that other SNPs lacked an association with specific breast cancer subtypes because we had limited samples and a lack of power to detect a true association. Larger sample sizes could help improve the power and ensure the correct conclusion regarding whether these SNPs are associated with specific breast cancer subtypes. Indeed, while this paper was in preparation, as part of the Southern China Breast Cancer Genetics Study, more participants are being recruited. We expect that the findings from the present study will be replicated.

Second, when we did analyses by receptor subtypes, 28.9% of cases were excluded due to unavailable information, which is similar to that reported by previous studies either conducted within the epidemiology registries [40] or conducted using receptor status measured at a single laboratory [41]. Furthermore, except the reporting hospital, comparison of demographic and clinical characteristics showed no significant difference between breast cancer patients included and excluded from present study. It is unlikely that the association between* FGFR2* SNPs and subtypes of breast cancers differed by whether corresponding information were available. Another weakness is that misclassification probabilities of subtypes are likely to be independent of susceptibly loci and thus would tend to underestimate association strengths rather than creating spurious associations [36]. For example, a recent study showed a high discordance between HER expression based on IHC and mRNA, 60% of the HER2+ by IHC tumors were not classified as HER2+ by mRNA [42]. To address these limitations, we are currently conducting a study aimed at evaluating the value of additional classifications to expand our understanding of the etiology of this heterogeneous tumor.

## 5. Conclusions

In conclusion, our study revealed a significant association of* FGFR2* intron-2 SNPs with breast cancer risk in Southern Han Chinese and provided strong evidence for differential susceptibility according to intrinsic subtype. Further epidemiological and experimental studies of larger datasets along with intrinsic subtype categorization are warranted to explore and confirm the role of these variants in increasing breast cancer risk, which will provide biological insights on the mechanisms of carcinogenesis and ultimately lead to improvement in prevention and treatment.

## Figures and Tables

**Table 1:** Characteristics of controls and breast cancer cases in the Southern China Breast Cancer Genetics Study.

Variables	Overall cases versus controls			Breast tumor subtypes				
	Controls (*n* = 882)	Cases (*n* = 609)	*P*	Luminal-B	Luminal-A	ER−HER2+	Triple negative	*P*
Age (years [mean ± SD])	45.2 ± 10.7	48.5 ± 9.5	**<0.001**	47.3 ± 9.7	48.0 ± 9.3	50.4 ± 9.2	49.1 ± 9.8	0.05
Body mass index (kg/m^2^ [mean ± SD])	23.6 ± 5.1	23.1 ± 5.0	0.07	22.7 ± 5.0	23.2 ± 5.3	23.0 ± 4.8	23.1 ± 5.0	0.77
Age at menarche (years [%])								
≤13	76.5	73.4	0.17	71.7	71.9	74.4	77.6	0.66
>13	23.5	26.6		28.3	28.1	25.6	22.4	
Nulliparous (%)	13.4	11.8	0.38	11.5	14.8	8.5	8.6	0.2
Age at first full-term pregnancy among parous women (years [%])								
≤30	97.2	86.4	**<0.001**	80.5	87.8	84.6	90.5	0.12
>30	2.8	13.6		19.5	12.2	15.4	9.5	
Menopausal status and HT (%)								
Premenopausal	33.9	33.7	**0.003**	28.3	38.8	28.2	32.8	0.06
Postmenopausal, never HT	8.8	14.9		23.0	10.6	19.7	12.1	
Postmenopausal, former HT	22.3	19.7		21.2	19.0	20.5	19.0	
Postmenopausal, current HT	34.9	31.7		27.4	31.6	31.6	36.2	

Abbreviations: SD, standard deviation; ER, estrogen receptor; HER2, human epidermal growth factor receptor 2; HT, hormonal therapy including estrogen therapy or estrogen plus progestin therapy.

**Table 2:** Minor allele frequency distribution in different ethnic groups from HapMap and Southern Han Chinese women.

SNP	Position	Allele (major/minor)	Minor allele frequency distribution					HWEP
			Present	HCB	JPT	CEU	YRI	
rs1078806	chr10: 121579461	T/C	28.8%	25.6%	26.2%	46.0%	23.0%	0.56
rs1219648	chr10: 121586676	A/G	45.3%	37.2%	31.2%	46.5%	46.4%	**<0.0001**
rs2420946	chr10: 121591810	C/T	39.5%	38.4%	31.4%	46.5%	59.8%	0.06
rs2981579	chr10: 121577821	C/T	44.9%	44.2%	37.2%	46.5%	67.3%	0.22
rs2981582	chr10: 121592803	C/T	34.9%	30.2%	23.3%	45.6%	51.3%	0.29

Abbreviations: HCB, Han Chinese in Beijing; JPT, Japanese in Tokyo; CEU, European descent from Utah; YRI, Yoruba individuals from Ibadan, Nigeria; HWEP, Hardy–Weinberg equilibrium *P* value.

**Table 3:** Association between the selected *FGFR2* polymorphisms and breast cancer risk according to intrinsic subtype.

Genotype or allele	Controls (%)	All breast cancer cases (*n* = 609)			Luminal-A cases (*n* = 263)			Luminal-B cases (*n* = 113)			ER−HER2+ cases (*n* = 117)			Triple negative cases (*n* = 116)		
	(*n* = 882)	Cases (%)	*P* value^a^	OR (95% CI)	Cases (%)	*P* value^a^	OR (95% CI)	Cases (%)	*P* value^a^	OR (95% CI)	Cases (%)	*P* value^a^	OR (95% CI)	Cases (%)	*P* value^a^	OR (95% CI)
rs1078806																
T/T	448 (51.1%)	293 (48.3%)	—	1.00	123 (47.1%)	—	1.00	52 (46.0%)	—	1.00	61 (52.1%)	—	1.00	57 (49.1%)	—	1.00
C/T	352 (40.2%)	260 (42.8%)	6.5 × 10^−1^	1.16 (0.93–1.44)	117 (44.8%)	3.6 × 10^−1^	1.24 (0.92–1.66)	47 (41.6%)	3.9 × 10^−1^	1.17 (0.77–1.78)	48 (41.0%)	3.6 × 10^−1^	1.06 (0.70–1.59)	48 (41.4%)	9.5 × 10^−1^	1.11 (0.74–1.68)
C/C	76 (8.7%)	54 (8.9%)	7.6 × 10^−1^	1.06 (0.72–1.56)	21 (8.1%)	8.5 × 10^−1^	0.97 (0.57–1.65)	14 (12.4%)	1.8 × 10^−1^	1.55 (0.82–2.94)	8 (6.8%)	4.3 × 10^−1^	0.73 (0.33–1.60)	11 (9.5%)	7.3 × 10^−1^	1.13 (0.57–2.27)
*P* trend			3.7 × 10^−1^			4.6 × 10^−1^			1.8 × 10^−1^			6.5 × 10^−1^			6.6 × 10^−1^	
C/T + C/C	428 (48.9%)	314 (51.7%)	2.2 × 10^−1^	1.14 (0.92–1.41)	138 (52.9%)	2.2 × 10^−1^	1.19 (0.90–1.57)	61 (54.0%)	2.8 × 10^−1^	1.24 (0.84–1.84)	56 (47.9%)	9.7 × 10^−1^	0.99 (0.67–1.47)	59 (50.9%)	5.9 × 10^−1^	1.11 (0.75–1.65)
T	1248 (71.2%)	846 (69.7%)	—	1.00	363 (69.5%)	—	1.00	151 (66.8%)	—	1.00	170 (72.6%)	—	1.00	162 (69.8%)	—	1.00
C	504 (28.8%)	368 (30.3%)	3.5 × 10^−1^	1.08 (0.92–1.27)	159 (30.5%)	4.7 × 10^−1^	1.08 (0.87–1.34)	75 (33.2%)	1.8 × 10^−1^	1.22 (0.91–1.63)	64 (27.4%)	6.8 × 10^−1^	0.94 (0.69–1.27)	70 (30.2%)	6.1 × 10^−1^	1.08 (0.80–1.45)
rs2420946																
C/C	323 (38.1%)	190 (31.5%)	—	1.00		—	1.00	35 (31.5%)	—	1.00	42 (35.9%)	—	1.00	40 (34.5%)	—	1.00
C/T	379 (44.8%)	297 (49.2%)	1.3 × 10^−2^	1.35 (1.07–1.71)	131 (50.6%)	**8.0** × **10** ^−**3**^	1.55 (1.12–2.15)	50 (45.0%)	3.7 × 10^−1^	1.23 (0.78–1.95)	63 (53.9%)	2.1 × 10^−1^	1.31 (0.86–2.01)	53 (45.7%)	5.7 × 10^−1^	1.14 (0.73–1.76)
T/T	145 (17.1%)	116 (19.2%)	4.7 × 10^−2^	1.36 (1.00–1.85)	55 (21.2%)	**1.1** × **10** ^−**2**^	1.69 (1.13–2.53)	26 (23.4%)	6.9 × 10^−2^	1.66 (0.96–2.86)	12 (10.3%)	1.9 × 10^−1^	0.64 (0.32–1.26)	23 (19.8%)	3.4 × 10^−1^	1.31 (0.75–2.27)
*P* trend			**2.1** × **10** ^−**2**^			**5.6** × **10** ^−**3**^			7.6 × 10^−2^			5.0 × 10^−1^			3.7 × 10^−1^	
C/T + T/T	524 (61.9%)	413 (68.5%)	**7.2** × **10** ^−**3**^	1.35 (1.08–1.69)	186 (71.8%)	**2.5** × **10** ^−**3**^	1.59 (1.17–2.16)	76 (68.5%)	1.6 × 10^−1^	1.35 (0.88–2.07)	75 (64.1%)	5.7 × 10^−1^	1.12 (0.75–1.69)	76 (65.5%)	4.2 × 10^−1^	1.18 (0.78–1.78)
C	1025 (60.5%)	677 (56.1%)	—	1.00	277 (52.5%)	—	1.00	120 (54.1%)	—	1.00	147 (62.8%)	—	1.00	133 (57.3%)	—	1.00
T	669 (39.5%)	529 (43.9%)	**2.0** × **10** ^−**2**^	1.19 (1.03–1.39)	241 (46.5%)	**5.0** × **10** ^−**3**^	1.32 (1.09–1.61)	102 (45.9%)	7.4 × 10^−2^	1.28 (0.98–1.68)	87 (37.2%)	5.4 × 10^−1^	0.92 (0.69–1.21)	99 (42.7%)	3.4 × 10^−1^	1.14 (0.87–1.50)
rs2981579																
C/C	275 (31.4%)	153 (25.2%)	—	1.00	68 (25.9%)	—	1.00	22 (19.6%)	—	1.00	31 (26.5%)	—	1.00	32 (27.6%)	—	1.00
T/C	415 (47.4%)	297 (48.9%)	4.0 × 10^−2^	1.30 (1.01–1.67)	118 (44.9%)	3.8 × 10^−1^	1.16 (0.83–1.63)	59 (52.7%)	2.5 × 10^−2^	1.80 (1.08–3.00)	64 (54.7%)	1.7 × 10^−1^	1.38 (0.87–2.18)	56 (48.3%)	5.5 × 10^−1^	1.15 (0.72–1.83)
T/T	186 (21.2%)	158 (26%)	**5.0** × **10** ^−**3**^	1.53 (1.14–2.05)	77 (29.3%)	**7.0** × **10** ^−**3**^	1.68 (1.15–2.45)	31 (27.7%)	**1.2** × **10** ^−**2**^	2.09 (1.17–3.73)	22 (18.8%)	9.1 × 10^−1^	1.03 (0.58–1.85)	28 (24.1%)	3.5 × 10^−1^	1.30 (0.75–2.24)
*P* trend			**3.8** × **10** ^−**3**^			**7.8** × **10** ^−**3**^			**1.2** × **10** ^−**2**^			7.3 × 10^−1^			3.5 × 10^−1^	
T/C + T/T	601 (68.6%)	455 (74.8%)	**8.1** × **10** ^−**3**^	1.37 (1.08–1.73)	195 (74.1%)	7.6 × 10^−2^	1.32 (0.97–1.81)	90 (80.4%)	**7.4** × **10** ^−**3**^	1.89 (1.16–3.08)	86 (73.5%)	2.8 × 10^−1^	1.27 (0.82–1.97)	84 (72.4%)	4.1 × 10^−1^	1.20 (0.77–1.85)
C	965 (55.1%)	603 (49.6%)	—	1.00	254 (48.3%)	—	1.00	103 (46.0%)	—	1.00	126 (53.8%)	—	1.00	120 (51.7%)	—	1.00
T	787 (44.9%)	613 (50.4%)	**4.0** × **10** ^−**3**^	1.24 (1.07–1.43)	272 (51.7%)	**8.0** × **10** ^−**3**^	1.29 (1.07–1.57)	121 (54.0%)	**1.1** × **10** ^−**2**^	1.43 (1.08–1.88)	108 (46.2%)	7.6 × 10^−1^	1.04 (0.79–1.37)	112 (48.3%)	3.4 × 10^−1^	1.14 (0.87–1.50)
rs2981582																
C/C	370 (43.2%)	238 (39.2%)	—	1.00	89 (34%)	—	1.00	41 (36.6%)	—	1.00	57 (48.7%)	—	1.00	51 (44%)	—	1.00
T/C	375 (43.8%)	266 (43.8%)	3.6 × 10^−1^	1.11 (0.89–1.40)	119 (45.4%)	7.1 × 10^−2^	1.33 (0.98–1.82)	48 (42.9%)	4.9 × 10^−1^	1.16 (0.75–1.81)	52 (44.4%)	6.8 × 10^−1^	0.92 (0.61–1.38)	47 (40.5%)	6.5 × 10^−1^	0.91 (0.59–1.39)
T/T	111 (13.0%)	103 (17.0%)	3.3 × 10^−2^	1.41 (1.03–1.94)	54 (20.6%)	**1.0** × **10** ^−**3**^	2.01 (1.35–3.01)	23 (20.5%)	3.3 × 10^−2^	1.83 (1.05–3.19)	8 (6.8%)	**4.3** × **10** ^−**2**^	0.45 (0.21–0.98)	18 (15.5%)	6.6 × 10^−1^	1.14 (0.64–2.04)
*P* trend			**3.1** × **10** ^−**2**^			**7.0** × **10** ^−**4**^			4.4 × 10^−2^			7.9 × 10^−2^			7.9 × 10^−1^	
T/C + T/T	486 (56.8%)	369 (60.8%)	1.2 × 10^−1^	1.18 (0.95–1.47)	173 (66%)	**6.7** × **10** ^−**3**^	1.49 (1.11–1.99)	71 (63.4%)	1.8 × 10^−1^	1.32 (0.88–1.99)	60 (51.3%)	2.8 × 10^−1^	0.81 (0.54–1.19)	65 (56.0%)	8.4 × 10^−1^	0.96 (0.65–1.42)
C	1115 (65.1%)	742 (61.1%)	—	1.00	297 (56.7%)	—	1.00	130 (58.0%)	—	1.00	166 (70.9%)	—	1.00	149 (64.2%)	—	1.00
T	597 (34.9%)	472 (38.9%)	**4.0** × **10** ^−**2**^	1.17 (1.01–1.36)	227 (43.3%)	**7.0** × **10** ^−**4**^	1.40 (1.15–1.71)	94 (42.0%)	5.0 × 10^−2^	1.32 (1.00–1.74)	68 (29.1%)	7.5 × 10^−2^	0.76 (0.57–1.03)	83 (35.8%)	8.6 × 10^−1^	1.03 (0.77–1.36)

^a^Adjusted for age, age at first full-term pregnancy, menopausal status, and hormonal therapy status.

Abbreviations: OR, odds ratio; CI, confidence interval; ER, estrogen receptor; HER2, human epidermal growth factor receptor 2.

**Table 4:** Frequencies of inferred haplotypes of* FGFR2 * (rs1078806, rs2420946, rs2981579, and rs2981582) in breast cancer cases and controls.

Haplotype^a^	Frequency			OR (95% CI)^b^	*P*
	Total (*n* = 1491)	Controls (*n* = 882)	Cases (*n* = 609)		
T-C-C-C	0.443	0.467	0.408	1.00	—
C-T-T-T	0.229	0.216	0.247	**1.30 (1.07**–**1.57)**	**8.1** × **10** ^−**3**^
T-T-T-C	0.066	0.059	0.076	**1.44 (1.04**–**1.99)**	**2.8** × **10** ^−**2**^
T-C-T-C	0.059	0.054	0.065	**1.42 (1.01**–**2.00)**	**4.5** × **10** ^−**2**^
T-C-C-T	0.057	0.052	0.064	1.38 (0.99–1.93)	5.9 × 10^−2^
C-T-T-C	0.046	0.047	0.045	1.14 (0.78–1.66)	5.0 × 10^−1^
T-T-T-T	0.045	0.043	0.047	1.25 (0.84–1.85)	2.8 × 10^−1^
T-T-C-T	0.019	0.021	0.015	0.89 (0.48–1.65)	7.1 × 10^−1^
C-C-T-C	0.014	0.018	0.009	0.63 (0.29–1.35)	2.4 × 10^−1^
Rare <1%	0.024	0.023	0.023	1.11 (0.64–1.95)	7.0 × 10^−1^

^a^Haplotype in the order of FGFR2 SNPs rs1078806, rs2420946, rs2981579, and rs2981582.

^b^Adjusted for age, age at first full-term pregnancy, menopausal status, and hormonal therapy status.

Abbreviations: OR, odds ratio; CI, confidence interval.
